# A Practice Combining Exercise and Social Interaction for Community-Dwelling Elderly People and Its Outcomes

**DOI:** 10.7759/cureus.76488

**Published:** 2024-12-27

**Authors:** Inoue Tadatoshi, Shogo Sawamura, Chinatsu Nakagawa, Tatsuya Sera, Takashi Nagai, Takahiro Takenaka, Kengo Kohiyama, Takashi Nakamura

**Affiliations:** 1 Department of Rehabilitation, Heisei College of Health Sciences, Gifu, JPN; 2 Elderly Welfare Section, Ito City Hall, Ito, JPN; 3 Faculty of Education, University of Teacher Education Fukuoka, Fukuoka, JPN

**Keywords:** community, dementia cafe, elderly, physical activity, preventive program

## Abstract

Introduction: The purpose of this study was to clarify the impact of an intervention combining exercise, lifestyle guidance, and community-building activities on the walking function of community-dwelling elderly individuals.

Methods: A total of 391 elderly participants (362 females, 29 males), aged 65 and above, were involved in a one-year intervention consisting of physical activities in a health exercise club, community-building activities, and dementia cafes. The walking function was assessed using an artificial intelligence (AI)-based gait analysis tool and health status was evaluated using a questionnaire. Pre- and post-intervention walking function and questionnaire scores were compared.

Results: After the intervention, walking speed improved, and both gait regularity and left-right asymmetry also showed improvements. The questionnaire scores also improved, and the number of frail elderly individuals decreased. Walking speed improved from 3.86 seconds to 3.09 seconds, and gait regularity improved from 6.96% to 5.75%.

Discussion: It is believed that muscle strength training and increased social interaction contributed to the improvement of walking function. Furthermore, community-based participatory activities played an important role in enhancing health status and psychological stability, suggesting an improvement in quality of life.

## Introduction

Japan's elderly population ratio reached 29.1% in 2023 and is projected to exceed 10 million by 2030, continuing to grow [1]. As a result, social security expenses are steadily increasing, making the sustainability of a sound social insurance system crucial. Furthermore, "Healthy Japan 21 (Third Basic Plan)" promotes the creation of environments in local communities that naturally enable people to maintain good health in order to extend healthy life expectancy, emphasizing the importance of individual health consciousness and social connections [2]. Ito et al [3]. conducted a preventive care program focusing on exercise with 45 community-dwelling elderly individuals and reported that both motor function and health-related quality of life improved after participation. In addition, Tanak et al [4]. conducted a survey on participation in community gathering places among 472 elderly individuals who were not certified as requiring long-term care and reported that participation in community gathering places had an effect of suppressing the risk of transitioning to a long-term care status. Regarding preventive programs for community-dwelling elderly individuals, it has been reported that interventions involving multiple factors, such as exercise and nutrition, are more effective than single interventions [5,6], indicating the importance of preventive programs in local communities. Previous reports have shown that preventive activities in local communities have been effective in interventions focusing on exercise or combining exercise with dietary support. However, there are limited reports detailing the progression of interventions that combine exercise, lifestyle guidance, and community engagement as part of a comprehensive approach. This study aimed to clarify how a comprehensive intervention combining exercise, lifestyle guidance, and community engagement influences health status and walking function.

## Materials and methods

Participants

Participant individuals aged 65 or older who participated in the health gymnastics club were included. Recruitment for the health gymnastics club was conducted through the city's website, postings at city offices and community centers, and referrals by staff at comprehensive community support centers and life support coordinators, as well as after health checkups. The principal investigator explained the purpose of the study to the city's project officer, and the officer recruited the participants. The principal investigator and the project officer also explained the purpose of the study to the participants, and those who consented to participate in the study were included. The health gymnastics club was held at 20 locations in the city for a period of one year. Participants who attended both the initial and final measurements were included in the analysis. Since this study included walking as a measurement item, the following criteria were set for the participants: 1. Living at home and not certified as requiring long-term care. 2. Exclusion criteria: Using a cane or other assistive device for mobility. Experiencing pain during walking. Requiring assistance with walking. Those who did not attend during the study period. Those with an attendance rate of less than 50%. The participants of this study were 391 (362 women, 29 men) with an average age of 79.36 ± 5.53 years. 

Measurement items

To comprehensively grasp the health status of the elderly, the Late-Elderly Questionnaire (hereinafter referred to as the "Questionnaire") was administered. The questionnaire consists of 15 questions covering health status, mental health, diet, oral function, changes in weight, exercise habits and history of falls, cognitive function, smoking history, social participation, and social support [7]. The response format for each question ranged from a two-point to a five-point scale, with question 1 scored as 0 for "good" or "fairly good" and 1 for "not very good" or "not good." Question 2 was scored as 0 for "satisfied" or "somewhat satisfied" and 1 for "somewhat dissatisfied" or "dissatisfied." Question 12 was scored as 0 for "never smoked" and 1 for "quit" or "currently smoking." The questionnaire was scored with 0 points for good health and 1 point for poor health, with a total score ranging from 0 to 15, indicating better health with lower scores. A score of 4 or more was defined as frailty [8].

For the assessment of gait function, a gait analysis system called Tolt (manufactured by Exa Home Care Co., Ltd.) was used. Comfortable gait at a normal speed was measured. The measurement was performed only once. Tolt is an application that analyzes gait using video footage captured by the participant, and its reliability and validity have been reported [9]. Tolt analyzes five meters of walking by calculating the rate of change in distance over time between the landmarks of the neck and the left and right hip joints displayed on the iOS device screen. The skeleton extraction used the original algorithm of ExaWizards Inc. (pose estimation and environment detection based on the OpenPose machine learning library) [10]. Tolt can evaluate "speed," "regularity," and "asymmetry" from recorded gait videos. In this study, the iPad was placed 150 cm above the floor to record the participant's gait. The placement was one meter behind the start line. The walking distance was 10 meters, and the central 5 meters was used as the measurement section. The speed was determined based on the size of the human body from the recorded gait. Regularity was determined by measuring the position of the left and right ankle joints and using the standard deviation of the swing phase time for each step. Asymmetry was determined by the ratio of the left and right stance phase times when the sum of the left and right stance phase times was 100 [11]. The evaluation was conducted by a team consisting of occupational therapists with more than a decade of experience in dementia care and a history of research, public health nurses, and staff from the senior welfare section involved in preventive care programs.

Intervention methods

The intervention consisted of three components: physical activity (health gymnastics, aerobic exercise, and strength training), community building, and dementia cafe. Health gymnastics were performed while seated in a chair, following music. Aerobic exercise in physical activity involves moving the body using a TheraBand or towel. Strength training aimed to improve upper and lower limb and balance functions, as well as posture and prevent falls. The frequency of sessions was once a week. Community building was initiated by local residents and involved light exercise, music, handicrafts, and other activities that people were good at while enjoying conversation. Sessions were held once a month for two hours. The dementia cafe, staffed by public health nurses, was a welcoming space for anyone to attend, from individuals with dementia and their families to local residents. Activities offered included dementia education, health talks, and opportunities for participants to connect with one another. Sessions were held once a month. The intervention period for this study was one year. Paired t-tests were used to compare the data before and after the intervention. The significance level was set at 5%. Data are presented as mean ± standard deviation. Analysis was performed using SPSS version 24 (IBM Corp., Armonk, New York, USA).

Ethical considerations

Informed consent was obtained from the participants after explaining the purpose of the study. This study was approved by the Ethics Review Board of Heisei Medical Junior College (Approval number: R02-04). There are no companies or other organizations with conflicting interests that should be disclosed in relation to this study.

## Results

In this study, we conducted a health gymnastics club for community-dwelling elderly individuals, which included physical activity, community building, and a dementia cafe. We compared the gait function and health and lifestyle scores before and after the intervention. As a result, walking speed improved from 3.86 ± 0.92 sec to 3.09 ± 1.06 sec, regularity improved from 6.96 ± 3.14 to 5.75 ± 2.57, asymmetry improved from 52.17 ± 1.94 to 51.56 ± 1.51, and the Questionnaire score improved from 2.25 ± 1.73 to 1.94 ± 1.66. The number of participants who scored four or more on the questionnaire and were classified as frail decreased from 83 to 55. The characteristics of the participants are shown in Tables 1, 2.

**Table 1:** Attributes of the participants (n=391). The table shows the minimum, maximum, mean, and standard deviation of attributes (age, walking speed, regularity, asymmetry, and questionnaire scores) for participants at baseline (X year) and after one year (X + 1 year). Regularity (standard deviation): Regularity measures the position of the left and right ankle joints and is the standard deviation of the time taken during the swing phase to install per step. Asymmetry (left/right ratio): The left-right difference is the ratio of the left-right difference when the time of the left-right stance period is added, and the ratio of the left-right difference is 100.

Attribute	Time	Minimum value	Maximum value	Mean	Standard deviation
Age		66	95	79.36	5.53
Speed (sec/5 m)	X year	2.2	9.1	3.86	0.92
Regularity (standard deviation)		0	19.6	6.96	3.14
Asymmetry (left/right ratio)		50	63	52.17	1.94
Health and lifestyle		0	9	2.25	1.73
Speed (sec/5 m)	X + 1 year	1.5	12.5	3.09	1.06
Regularity (standard deviation)		0	20	5.75	2.57
Asymmetry (left/right ratio)		50	59.4	51.56	1.51
Health and lifestyle		0	13	1.94	1.66

**Table 2:** Comparison of walking function and questionnaire results before and after the intervention (n=391). Mean ± SD, paired t-test. Regularity (standard deviation): Regularity measures the position of the left and right ankle joints and is the standard deviation of the time taken during the swing phase to install per step. Asymmetry (left/right ratio): The left-right difference is the ratio of the left-right difference when the time of the left-right stance period is added, and the ratio of the left-right difference is 100.

Attribute	X year	X + 1 year	p-value
Speed (sec/5 m)	3.9 ± 0.9	3.1 ± 1.1	<0.01
Regularity (standard deviation)	7.0 ± 3.1	5.8 ± 2.6	<0.01
Asymmetry (left/right ratio)	52.2 ± 1.9	51.6 ± 1.5	<0.01
Health and lifestyle	2.3 ± 1.7	1.9 ± 1.6	<0.01

## Discussion

In this study, a health exercise club was implemented for community-dwelling elderly individuals, focusing on physical activity, community building, and a dementia cafe. The results showed an improvement in health and lifestyle scores, as well as increased walking speed, regularity, and symmetry. Additionally, the number of individuals classified as frail decreased.

One of the key factors contributing to the improved health status, psychological state, dietary habits, exercise habits, and cognitive function of elderly participants in the city-organized health classes is that these classes serve as a social gathering place. For the elderly, feelings of isolation and social exclusion can lead to psychological distress and depression. However, participation in health classes and dementia cafes fosters social interaction and a sense of belonging to the community, thereby reducing stress and improving depressive symptoms. Honda et al. reported that frequent participation in elderly gatherings has an inhibitory effect on depression [12]. Furthermore, Kimura et al. stated that a musculoskeletal improvement program in a care prevention class for community-dwelling elderly individuals promoted social activities and participation, highlighting the importance of preventive activities in the community [13].

Health classes also provided guidance on nutrition and the importance of exercise habits. Learning about the importance of a balanced diet and incorporating it into daily eating habits, as well as ensuring three meals a day, can improve overall health, prevent nutritional deficiencies, and contribute to improved overall physical function. Kawabata et al. reported that implementing a multi-component program targeting exercise, nutrition, and social participation for community-dwelling older adults improved homebound status and dietary habits [14]. As shown above, creating a sense of community and offering various programs in health classes can enhance participants' health and psychological well-being by fostering social interaction, improving dietary habits, establishing exercise habits, and providing cognitive stimulation. These elements are interconnected and contribute comprehensively to improving the quality of life for the elderly.

Next, we will discuss walking. One reason for the improvement in the three walking parameters is the increased muscle strength throughout the body due to aerobic exercise and strength training. Lower extremity muscle strength is closely linked to walking speed and balance ability, and it has been reported that strength training can significantly enhance walking ability [15,16]. Additionally, a systematic review by Keating et al. stated that strength training improves walking speed and balance in the elderly and contributes to increased activity in daily life, indicating the importance of strength training for improving walking function [17]. Similarly, in the participants of this study, overall muscle strength increased through strength training. In particular, lower extremity muscle strength is one of the determining factors of walking speed. As muscle strength increases, the force with which the ground is pushed off increases, leading to a wider stride and improved walking speed. Moreover, improved core muscle strength facilitates posture maintenance during walking, resulting in more stable walking and improved regularity and symmetry. We believe that participation in community building and dementia cafes increased the frequency of going out and physical activity, which contributed to the improvement in walking speed, regularity, and symmetry. Taniguchi et al. [18] stated that an increase in physical activity is related to improved walking function, and Abe et al. [19] reported that there is a close relationship between activity levels and physical functions necessary for mobility, indicating the importance of ensuring daily activity levels for maintaining and improving walking function.

Limitations and future directions

In this study, we conducted a pre-post comparison of the intervention within the context of a government-led initiative, but we were unable to establish a control group. In future studies, it is necessary to set up a control group to verify the effectiveness of the intervention. While we conducted a comprehensive assessment of the health status of older adults using a questionnaire, detailed evaluations of individual items were not possible. Additionally, due to the large number of venues, it was difficult to conduct detailed evaluations. However, by increasing staff in the future, we can conduct more detailed evaluations and clarify the life challenges faced by the participants. Due to the large number of venues and participants and insufficient manpower, we were unable to record the number of times each participant attended. In the future, it will be necessary to keep attendance records to examine the participation rate and the effects of the intervention.

## Conclusions

This study conducted a health exercise club for one year targeting community-dwelling elderly individuals, focusing on physical activity, community building, and a dementia cafe. Improvements were also observed in health status, walking function, and a reduction in the prevalence of frailty. Furthermore, providing opportunities for social interaction reduced feelings of isolation, promoted psychological stability, and increased the frequency of outings. These results suggest that preventive activities in the community are important for maintaining health and improving the quality of life of the elderly.
